# Gender Recognition Based on Gradual and Ensemble Learning from Multi-View Gait Energy Images and Poses

**DOI:** 10.3390/s23218961

**Published:** 2023-11-03

**Authors:** Tak-Man Leung, Kwok-Leung Chan

**Affiliations:** Department of Electrical Engineering, City University of Hong Kong, Hong Kong, China; takmleung2-c@my.cityu.edu.hk

**Keywords:** gender recognition, gait energy image, posture, walking cycle, cascade network, ensemble learning

## Abstract

Image-based gender classification is very useful in many applications, such as intelligent surveillance, micromarketing, etc. One common approach is to adopt a machine learning algorithm to recognize the gender class of the captured subject based on spatio-temporal gait features extracted from the image. The image input can be generated from the video of the walking cycle, e.g., gait energy image (GEI). Recognition accuracy depends on the similarity of intra-class GEIs, as well as the dissimilarity of inter-class GEIs. However, we observe that, at some viewing angles, the GEIs of both gender classes are very similar. Moreover, the GEI does not exhibit a clear appearance of posture. We postulate that distinctive postures of the walking cycle can provide additional and valuable information for gender classification. This paper proposes a gender classification framework that exploits multiple inputs of the GEI and the characteristic poses of the walking cycle. The proposed framework is a cascade network that is capable of gradually learning the gait features from images acquired in multiple views. The cascade network contains a feature extractor and gender classifier. The multi-stream feature extractor network is trained to extract features from the multiple input images. Features are then fed to the classifier network, which is trained with ensemble learning. We evaluate and compare the performance of our proposed framework with state-of-the-art gait-based gender classification methods on benchmark datasets. The proposed framework outperforms other methods that only utilize a single input of the GEI or pose.

## 1. Introduction

Gender classification is a task in which humans excel. If the gender of a human can be recognized automatically by a machine, it will be very helpful in many applications such as intelligent surveillance, micromarketing, etc. Information on the gender of visitors in the crowd flow is of great commercial value for better shop arrangement and allocation, better promotion management, and human flow arrangement. Foggia et al. [1] illustrated many examples, such as personalized advertising according to soft biometrics. They proposed a multi-task network for the recognition of various biometrics, including gender. Automatic gender recognition also plays an important role in human–machine interaction and security control.

Some methods have been proposed to tackle the gender classification problem with the use of non-visual biometric techniques such as voice recognition. In practical applications, it is more convenient to acquire visual information. For instance, the gender of the subject is recognized using the image of depth data [2]. The human gender is predicted by the regression model with the input of 3D coordinates of 20 joints. To extend the viewing range, Guffanti et al. [3] proposed a multiple-depth camera gender classification system with the use of two depth sensors. Various temporal and spectral features are computed from the 3D coordinates of 25 joints. The human gender is recognized with the support vector machine (SVM) classifier.

Another biometric technique is face recognition. Greco et al. [4] proposed a computer vision system that can recognize the gender of the person from the face image captured in an unconstraint scenario. Duan et al. [5] proposed a convolutional neural network (CNN) to extract facial features from the input images. The intermediate features are then input to a fully connected neural network for gender prediction. Fang et al. [6] proposed a gender classification framework with multi-stage learning. The first stage is an encoder-decoder network that performs human–background segregation. The second stage, based on VGG-19, is trained for gender prediction. For gender classification, high-resolution face images are often used. However, humans may not be cooperative, or their faces are occluded. In other system setups, e.g., a surveillance camera is used to capture the human. There is a long distance between the camera and the humans of interest. In all these cases, the camera may not capture full-face images, or the acquired image is of low resolution. It is very difficult, if not impossible, to extract useful facial features for gender classification. Facial images also have the problem of small inter-class and large intra-class differences.

Gait is useful visual information. Even from a long distance, the posture of the subject can still be perceived in the image. Therefore, gait-based gender recognition is a feasible approach. The gait of a human shows her/his posture and also their walking style. Amsaprabhaa et al. [7] presented a survey on a gait analysis framework based on visual input. Besides gait recognition methods, they also provided detailed insight into gait databases and performance evaluation metrics. Many research studies [8] adopt the gait-based approach, e.g., activity recognition, tracking, person identification, gender classification, etc. Gait can be recognized based on structural information such as stride parameters. Liao et al. [9] proposed a gait recognition method based on a 3D human pose estimated from images with a CNN. This type of method usually demands an initialization phase, such as model construction and the optimization of a large number of free parameters. Alternatively, methods can purely rely on appearance features extracted from the image. This type of method has the advantage of lower computational cost. For instance, in each image, the gait silhouette is segmented. The feature is then computed from the silhouette sequence, e.g., gait energy image (GEI) [10]. The GEI, computed as an average silhouette image, characterizes the movements of the subject over a gait cycle. However, the classification accuracy may be low on image sequences acquired at some viewing angles due to the highly similar GEIs of both gender classes. Moreover, the average silhouette does not display the posture of the subject clearly. Poses at distinctive phases of the walking cycle are useful for gender classification. We, therefore, propose a multi-stream cascade gender classification framework with the inputs of GEIs and poses extracted from the walking cycle. Based on the proposed framework, we design three models for gender recognition. For each model, the feature extractor network gradually learns from a variety of inputs. The multiple features are fed to the classifier network, which is trained by ensemble learning. We train our proposed models on benchmark gait datasets. We demonstrate that our gender classification models achieve higher accuracy than other methods that only utilize a single input. The contributions of our work are as follows:

- The GEI provides a concise representation of movement that can be used for gender classification. However, the GEI lacks photometric information and does not clearly display body shapes. We observe that postures, such as stance and swing images of the walking cycle, exhibit unique features that can provide complementary information for gender classification. In order to improve the gender classification accuracy, we exploit multiple modality inputs of the GEI and postures.
- We propose a multi-stream network for feature extraction from the multiple modality inputs. The extracted features are fused and fed to the classifier. We design the training process to allow the feature extractor network to gradually learn from a variety of inputs. The proposed cascade framework, through ensemble learning, predicts the gender class irrespective of other factors such as viewing angle and walking status.
- We adopt data augmentation to address the class imbalance problem of the gait dataset. An investigation is performed on the CASIA B and OU-ISIR MVLP datasets. Comparison analysis is carried out with recently proposed methods based on deterministic and deep learning approaches. We demonstrate that our proposed models outperform these reference methods that only utilize either the GEI or posture image.

The rest of this paper is organized as follows. The related research on gait recognition is reviewed in Section 2. We focus on the gait-based gender recognition techniques and the datasets created for gender classification research. Section 3 explains in detail our proposed gender classification framework. Based on the proposed framework, we design three gender classification models for comparison experiments. We train and test our proposed models on publicly available gait datasets. Some quantitative measures are adopted for performance evaluation. In Section 4, we compare the performance of our proposed framework with other methods. Section 5 presents the ablation studies performed on our proposed gender classification models. Finally, we conclude our investigation and suggest some future work in Section 6.

## 2. Related Work

Gait recognition methods, depending on the method of extracting visual information from the images, can be grouped into two categories—deterministic algorithm and deep learning-based model.

### 2.1. Deterministic Algorithm

Luo and Tjahjadi [11] proposed a gait recognition and understanding system based on the 3D human body pose. Semantic 3D features are estimated from 2D gait images. Gait states, such as view angle and walking status, are recognized by a sequence network. The deterministic algorithm performs gender classification via the computation of hand-crafted features, followed by gender prediction from the feature vector. Kovač et al. [12] utilized wavelet transform to extract gait features. In [13], the textural feature, local binary pattern (LBP), is computed from GEIs and then input to the classifier. SVM is a popular choice of classifier in many recently proposed methods, e.g., [13], due to its robustness. Saini and Singh [14] proposed a gender recognition system using SVM and multi-linear discriminant analysis (MDA) as classifiers. Do et al. [15] proposed a view-dependent gender classification system. The viewing angle (i.e., the walking direction) is first estimated. The gender of the human captured in arbitrary view is predicted with multiple view-dependent SVM classifiers.

### 2.2. Deep Learning Model

Deep learning brings forth rapid advancement in computer vision. In contrast to deterministic algorithms, deep learning is machine learning based on learning data representations. With the development of CNNs and the use of graphics processing units (GPUs), significant advancement has been reported. The CNN model is trained to learn feature extraction with the use of a training dataset. In many research studies, it is found that features extracted by deep learning-based algorithms can vastly outperform hand-crafted features computed by deterministic algorithms. Gait recognition also benefits from the adoption of the CNN model. Li et al. [16] proposed a CNN model to extract spatio-temporal features from the key frames of a gait sequence. Alternatively, Gul et al. [17] proposed a 3D CNN model to capture spatio-temporal features directly from the gait sequence. Dong et al. [18] proposed a gait recognition framework based on multiple input signals. They experimented with a number of algorithms, e.g., SVM and CNN, as the classifier. Wen et al. [19] proposed a multi-view gait recognition model based on a generative adversarial network (GAN). The generator network is used to transform the gait image of other views to normal views. Finally, recognition results of the normal views are fused. Deep learning models heavily rely on loss functions. Zhang et al. [20] proposed a robust gait-based loss function for gait recognition. They utilized CNN and long short-term memory (LSTM) units to extract spatio-temporal gait features. Luo and Tjahjadi [21] also exploited the LSTM deep network for spatio-temporal feature extraction in their proposed 3D gait recognition model.

Shiraga et al. [22] developed a gait recognition method from GEIs with the use of CNN for human identification. Sakata et al. [23] first proposed a network for classifying gender, age group, and age from GEIs. It contains one convolutional block and three parallel fully connected layers. They further proposed a larger network to address the same classification problem. It contains 13 structurally identical convolutional blocks organized into three layers. Xu et al. [24] proposed a CNN framework for real-time gender classification. From a single image, the human silhouette is segmented by graph cuts. Based on the single silhouette, a gait cycle is synthesized by the phase-aware reconstructor [25]. The gait cycle is then input to GaitSet [26] for gait recognition. In training, two images from two viewing angles are selected. The two synthesized gait cycles are fed into two GaitSet networks for model learning. In the testing phase, only one image frame is used as input, and one GaitSet is utilized as the feature extractor. The method has the advantage of quick response since recognition is performed per image instead of waiting for the acquisition of the whole gait cycle. The GEI, containing a mixture of static and dynamic gait features from the original image sequence, provides distinctive features for gait recognition. Features extracted from the synthesized gait cycle will be less precise. Moreover, the complex framework with two networks demands a high computational load in training. Besides the GEI, some state-of-the-art methods exploit the pose information for gait recognition. Kwon et al. [27] proposed the joint swing energy, which is computed from the skeletons found in three coordinate planes. Zhao et al. [28] utilized a pre-trained convolutional network to generate the pose heatmap from RGB images.

Besides using the single input of the GEI, some research utilizes additional input information and adopts a multi-stream framework for gender classification. Bei et al. [29] proposed a two-stream CNN to combine GEIs and optical flow information. A single GEI lacks temporal information on the walking cycle. To address this limitation, they proposed a new average silhouette subGEI, which is computed from fewer image frames than the complete gait cycle. Temporal information, characterized by the optical flow map, is then computed from two adjacent subGEIs. Russel and Selvaraj [30] proposed a unified model with the input of GEIs to six parallel CNNs. The parallel network contains a varying number of convolution layers. Each GEI is transformed into a multi-scale representation for the network to learn the discriminative gait features.

Multi-stage architecture has attracted researchers in recent years. Methods have been proposed to address various image-processing applications. For instance, Fu et al. [31] proposed a multi-stage network for image restoration. One approach to train complex multi-stage networks is via ensemble learning. The ensemble learning method, with the use of multiple learning algorithms, can enable the multi-stage network to produce better predictions than a simpler network trained with only one learning algorithm. Sethi et al. [32] developed an ensemble model based on CNN and LSTM for gait analysis. The inputs are skeleton and landmark data, which must be estimated first from the image. Anbalagan and Anbhazhagan [33] proposed an ensemble deep learning strategy for gait classification. Gait images are fed to two classifiers, the multi-layer perceptron and deep neural network. The scores are finally input into the LSTM unit for final classification. There are drawbacks to recurrent networks like LSTM. They are designed for language tasks. Their structures are sometimes not robust enough to process image data. Recurrent networks also suffer from the problem that gradients are prone to disappear or explode. Therefore, our proposed framework only utilizes multiple CNNs. Mogan et al. [34] proposed a multi-model gait recognition system based on CNN and transformer. The GEI is input to three models. The final classification is performed by averaging the three prediction scores. However, the computation cost of a transformer block is higher than that of a convolutional block. Transformer-style models are computationally more expensive than CNN models. As for our proposed framework, we adopt a different training strategy for the Stage 1 and Stage 2 CNN. To the best of our knowledge, our work is the first to adopt ensemble learning for training the gait-based gender classification method. The cascade framework contains two CNNs, each being trained with a different algorithm. The first-stage networks extract representative features from multiple inputs. Instead of fusing the prediction scores of first-stage networks, our framework fuses the extracted features and then feeds them to the second-stage network for further analysis. The second-stage network performs gender classification based on the fused feature vector. This approach provides more analytical capability and eventually enables the framework to produce more accurate gender predictions.

### 2.3. Gait Dataset

In order to facilitate the development of a data-driven gait recognition model, various gait datasets have been created. An accurate CNN model demands training on a large dataset. For instance, gait databases should contain videos capturing a large number of human subjects. Each subject should be instructed to walk in different directions and/or recorded by multiple cameras set in a wide range of viewpoints. Sakata et al. [23] trained their frameworks on a single-view gait dataset OU-ISIR LP [35]. OU-ISIR LP is a large dataset containing 32,753 females and 31,093 males with a wide range of ages. The videos were captured by a single camera. Moreover, the dataset provides the pre-computed GEIs. Xu et al. [24] trained their model on a multi-view gait dataset OU-ISIR MVLP [36]. OU-ISIR MVLP contains videos captured by 14 cameras set in different view angles. The dataset also provides a total of 267,386 pre-computed GEIs. Zhang and Wang [37] created a small gait dataset IRIP Gait Database. It only contains 32 male subjects and 28 female subjects. The Soton database [38] is a relatively old dataset. It contains 400 subjects with unique multi-modal data (e.g., multi-view gait records and face images). CASIA [39] is also a gait database with multi-view images. Another large-scale dataset, GREW [40], was created for research on gait recognition in the wild. It provides silhouette sequences, GEIs, and optical flow maps computed from videos captured from 26,345 subjects.

## 3. Gender Classification Framework

**Figure 1:**
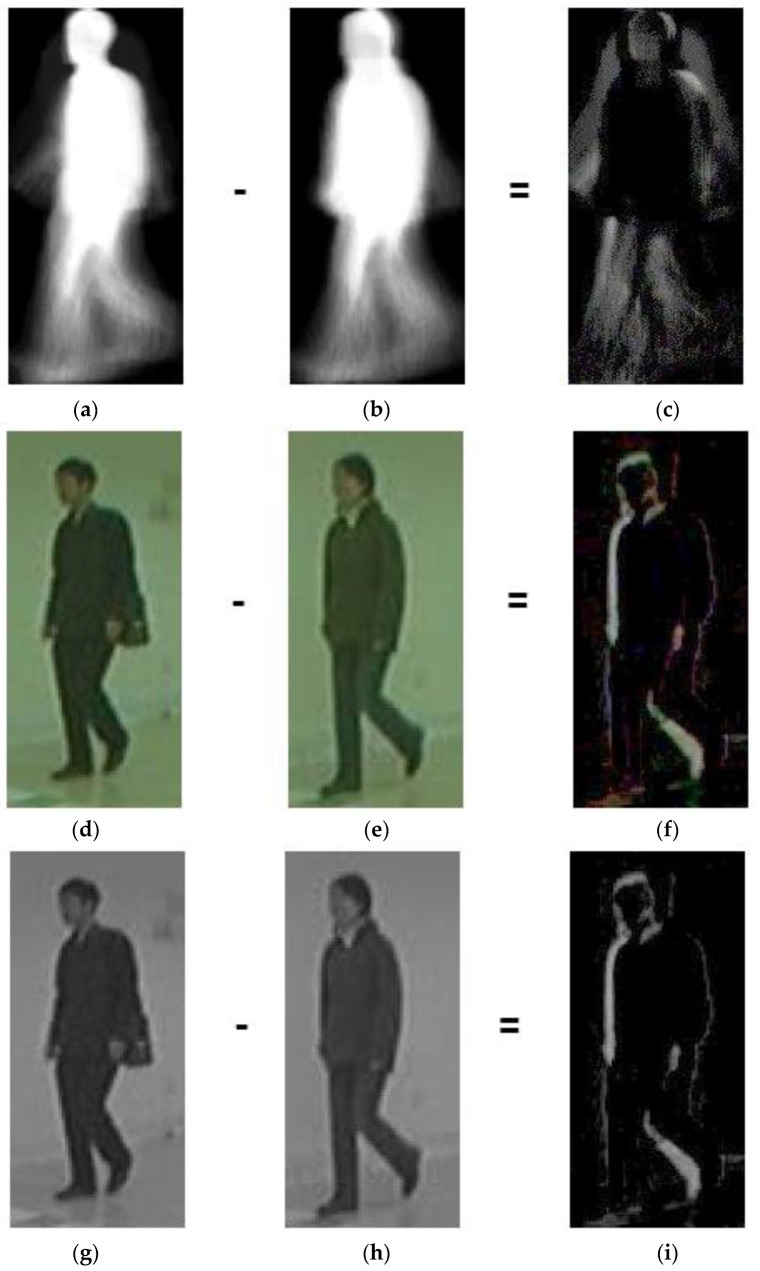
Differences in male and female GEIs and posture images: (**a**) male GEI; (**b**) female GEI; (**c**) difference between male and female GEIs; (**d**) male RGB image; (**e**) female RGB image; (**f**) difference between male and female RGB images; (**g**) male grey-scale image; (**h**) female grey-scale image; and (**i**) difference between male and female grey-scale images. The difference images are enhanced by gamma correction with the same factor of 0.5 for better visualization.

The GEI, which is computed from the silhouette images over a gait cycle, concisely represents the movement of the subject. Figure 1a,b show the GEIs of a male and a female, respectively. To observe the difference between the two GEIs, we perform image subtraction and enhance the difference of GEIs with gamma correction for better visualization, as shown in Figure 1c. The brighter pixels in the subtraction result correspond to a larger difference between the two GEIs. Gender recognition based on GEIs learns from the dissimilarity between male and female GEIs in gender prediction. Besides the exploitation of movement information, we propose a framework that also learns the dissimilarity between male and female postures. Figure 1d,e show the RGB images of a male and a female at the same phase of the walking cycle, respectively. The subtraction result, as shown in Figure 1f, illustrates the difference in the postures. This phenomenon is also observed from grey-scale images of males and females, as shown in Figure 1g–i. It is clear that the dissimilarity of postures can provide complementary information to that from the dissimilarity of GEIs. The gender recognition model can learn other features from posture images and benefit gender classification. In order to improve the gender recognition accuracy, we, therefore, propose a multi-stream gender classification framework with the inputs of GEIs and the grey-scale stance and swing images extracted from the walking cycle.

**Figure 2:**
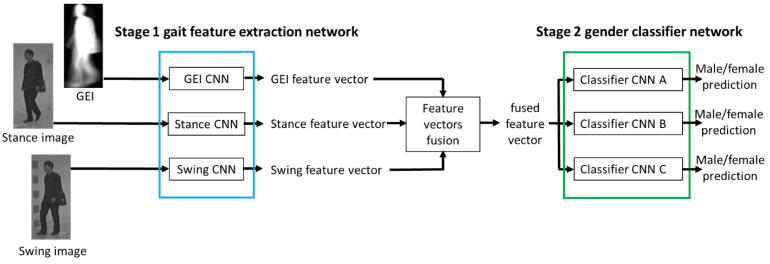
Proposed gender classification framework.

Figure 2 shows the proposed gender classification framework. Stage 1 contains three parallel streams of CNN, which are trained to extract gait features from the input images. The first input is the GEI computed from the silhouette sequence. The second and third inputs are the stance and swing images extracted from the walking cycle video. The feature vectors extracted by the CNNs are concatenated. Ensemble learning is adopted to train the Stage 2 CNN for gender prediction. The Stage 1 CNN is trained to extract features from multiple gait images (GEIs and stance and swing walking poses). After getting all the dense features from multi-sources, they are fused and input to the Stage 2 CNN. For the classifier network, we propose three CNNs (Stage 2A, Stage 2B, and Stage 2C) with different structures. The complete framework is further trained to extract effective features holistically. Through the injection of supervision at each stage, this gradual learning/incremental learning approach enhances the gait feature representation and inferencing capability of our proposed gender classification framework. Based on the proposed framework, we design three gender classification models, which are described in the following sections.

### 3.1. Gait Feature Extraction from GEI

The GEI is an appearance-based gait feature. As shown in Equation (1), it is computed as the weighted average values of the aligned human silhouettes. First, a human silhouette is detected in each image frame of the video capturing the subject walking, either manually or automatically by a background subtraction method. With the size of the human silhouette in each image frame normalized, a silhouette sequence is generated. The period of the walking cycle *N* is estimated. Finally, the GEI, in the format of a grey-scale image, is computed by averaging a number of image frames of silhouette sequence *S*:

(1) $$GEI\left(x,y\right)=\frac{1}{N}\sum_{t=1}^{N}S_{t}(x,y)$$

where *S_t_*(*x*, *y*) is the pixel value of the image frame of silhouette sequence *S* at coordinates (*x*, *y*) at time instant *t* of the gait cycle. The GEI is a concise representation of the motion of the human subject.

**Figure 3:**
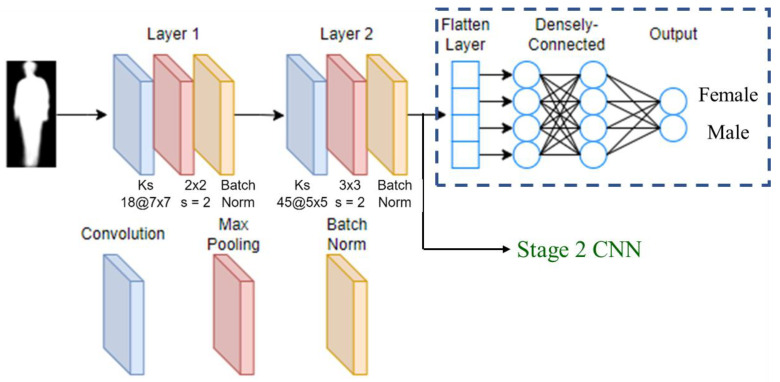
GEI CNN model.

We design a CNN for gait feature extraction from the input of the GEI. As shown in Figure 3, the GEI CNN model consists of convolutional layers, max-pooling layers, and batch normalization layers. If we use the single-stage network for gender classification (e.g., in the ablation study, which is explained in Section 5), a densely connected layer and an output layer are added. For the cascade gender classification framework, the feature vector extracted from the GEI will be fused with the other feature vectors and fed into the Stage 2 CNN. Therefore, for single-stream Stage 1 (only GEI CNN), the feature from Layer 2 is forwarded to a fully connected network for gender classification. For a complete framework (three-stream Stage 1 + Stage 2), features from the three-stream Stage 1 network are fused and then forwarded to the Stage 2 gender classification network.

**Figure 4:**
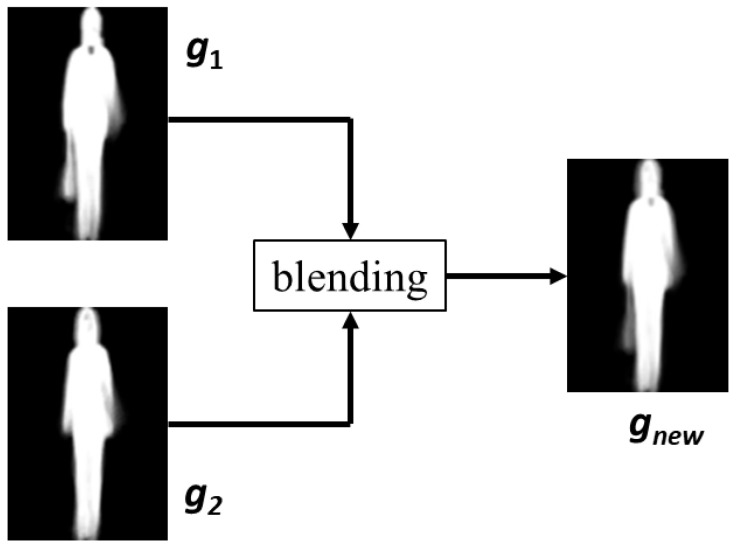
Generation of augmented female GEI.

There is an imbalance problem in the gait dataset. For instance, the CASIA B gait dataset [39] contains 10,187 male GEIs and 3405 female GEIs. To address this problem, we adopt data augmentation to increase the number of female GEIs to the same as their male counterparts. First, a new female GEI (*g_new_*) is generated by blending two original female GEIs (*g*_1_, *g*_2_), as shown in Figure 4. The blending function performs a cross-dissolve between *g*_1_ and *g*_2_

(2) $$g_{new}=\left(1-\alpha \right)g_{1}+\alpha g_{2}$$

where *α* is set to 0.5. A total of 45,559 new female GEIs are generated, from which 6782 are randomly selected as the augmented female data. All the synthesized female GEIs are included in the training set. The validation and test sets have no synthesized female GEIs.

### 3.2. Gait Features Extraction from Stance and Swing Images

The gait of human walking is periodic. Within one walking cycle, one foot stays on the ground (stance phase) for about 60% of the cycle and then lifts off the ground (swing phase) for about 40% of the cycle. We propose the gender classification framework, which also extracts posture-related features from image sequences. The grey-scale images of the stance and swing phases of the right leg are manually selected from the video. To address the imbalance problem, more samples are selected from the female videos. Each video contains many image frames capturing multiple walking cycles of the subject. We can sample more stance and swing image frames from each video, and thus, there is no need to utilize the blending method as in GEIs to obtain more female images. The dataset contains 10,177 male stance images, 10,177 male swing images, 6944 female stance images, and 6944 female swing images. The stance and swing images are fed into the respective CNN models, as shown in Figure 2. The Stance CNN and Swing CNN models have the same structure as the GEI CNN model (see Figure 3).

### 3.3. Gender Prediction CNN

**Figure 5:**
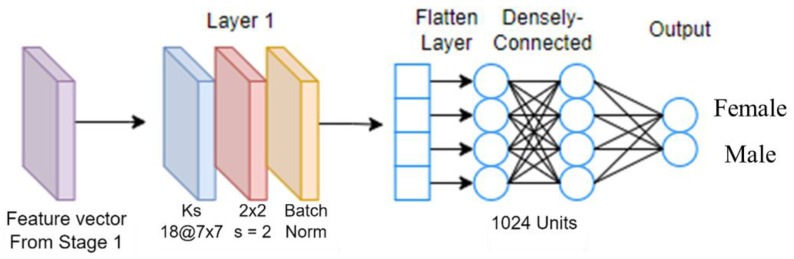
Stage 2A CNN model.

The feature vectors (each has a dimension of 1 × 1024) extracted by the three CNN models, as mentioned previously, are fused to form a wide feature vector of 1 × 3072. It is then reshaped to 128 × 24 and input to the Stage 2 CNN. Ensemble learning is adopted to train the CNN for the final gender prediction output. We propose three versions of the Stage 2 CNN. Figure 5 shows the one-layered model Stage 2A. It consists of a convolutional layer, a max-pooling layer, a batch normalization layer, a densely connected layer, and an output layer. That shallow network has the advantage of faster run time. The densely connected layer contains more nodes. The network has more trainable parameters than the other two Stage 2 CNNs. As illustrated in our ablation study, its gender classification accuracy can be as good as other Stage 2 models.

**Figure 6:**
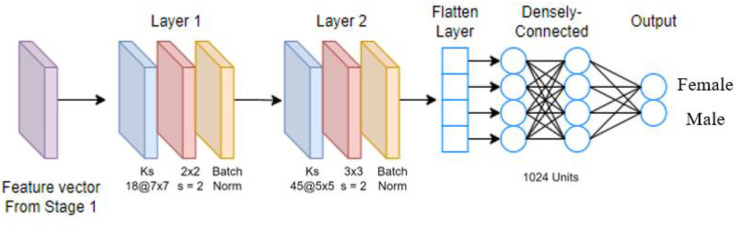
Stage 2B CNN model.

**Figure 7:**
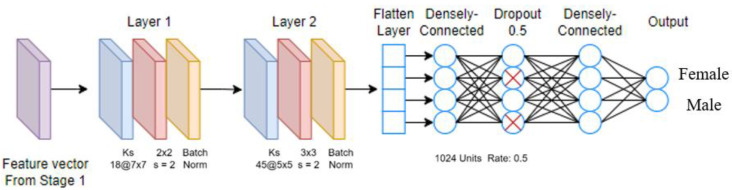
Stage 2C CNN model.

Figure 6 shows the two-layered model Stage 2B. There are two convolutional blocks, each containing a convolutional layer, a max-pooling layer, and a batch normalization layer. As compared with the Stage 2A model, it has more computation layers. Figure 7 shows the two-layered-plus-dropout model Stage 2C. There are two densely connected layers, and in between, we insert a dropout layer. As compared with Stage 2B, the dropout layer provides a larger variety of network structures during the training process.

### 3.4. Training Process

We adopt the Adam optimizer with a learning rate of 0.001. Besides the learning rate, another hyperparameter is the dropout rate of the Stage 2C CNN model. The gender classification framework is trained in two steps. First, individual Stage 1 CNNs are trained using the Leave-One-Sample-Out (LOSO) method. Each Stage 1 CNN is trained to predict the gender class irrespective of other factors such as viewing angle and walking status. Two models with the lowest loss and highest accuracy will be shortlisted for further testing. The best Stage 1 CNNs are fused and joined with the Stage 2 CNN. In the second step of training, the complete cascade framework (Stage 1 + Stage 2 CNNs) is trained with an 80/20 split of the dataset to predict the gender class.

**Figure 8:**
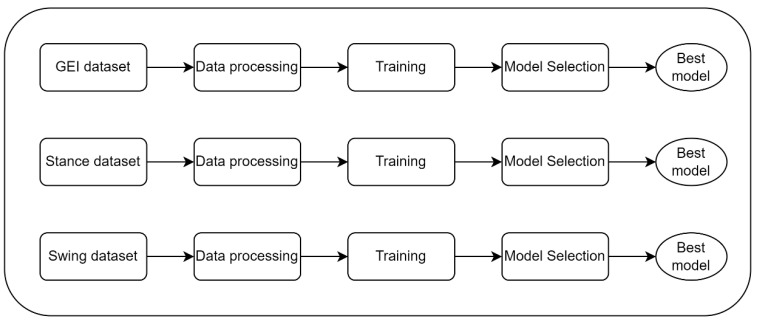
Overview of Stage 1 CNN optimization.

**Figure 9:**
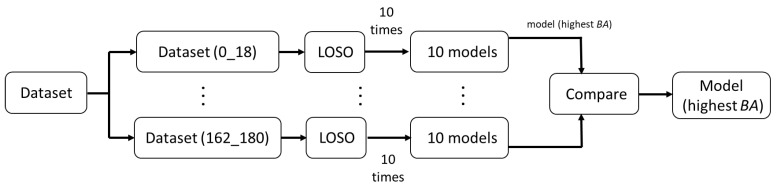
Training of Stage 1 CNN using LOSO.

The GEI CNN model, Stance CNN model, and Swing CNN model are trained independently, as shown in Figure 8. The CASIA B dataset [39] contains videos captured at 11 viewing angles. In the data processing step, inputs from two different angles are combined to form one set of samples. All combinations of two viewing angles are used to train the feature extractor network. As shown in Appendix A, a total of 55 sets are generated (e.g., 0° + 18° is the set containing inputs from viewing angles of 0 and 18 degrees). This training process ensures the Stage 1 network is optimized to extract gait features from multi-modality inputs in multiple viewing angles. The better gender classification results of our proposed model over all other methods demonstrate the effectiveness of our proposed feature extractor and classification networks. As shown in Figure 9, LOSO is adopted as the training method, which is repeated 10 times for each training set. The total training time is about 1100 h.

**Figure 10:**
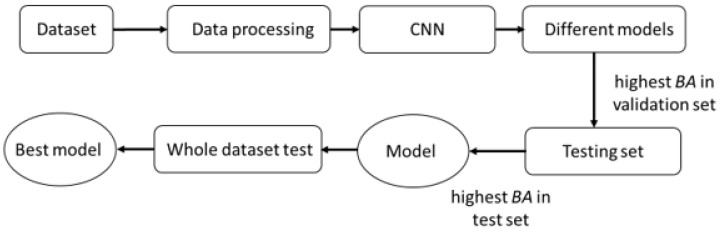
CNN model selection.

Figure 10 shows the selection of the CNN model. Trained models with the highest balanced accuracy are further tested. The model with the highest balanced accuracy in the test set is finally used to perform gender classification on the whole gait dataset. We adopt categorical cross entropy *CE* as the loss function

(3) $$CE\left(x\right)=-\sum_{i=1}^{C}y_{i}log(f(x_{i}))$$

where *y_i_* is the ground truth label for gender class *i*, *x_i_* is the score for gender class *i*, *f* is the activation function sigmoid, and *C* is the number of gender classes.

**Figure 11:**
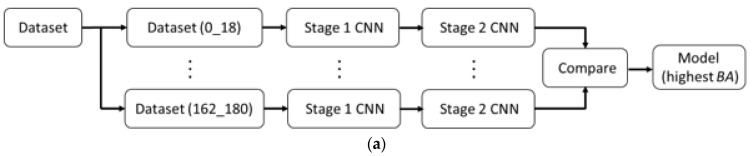

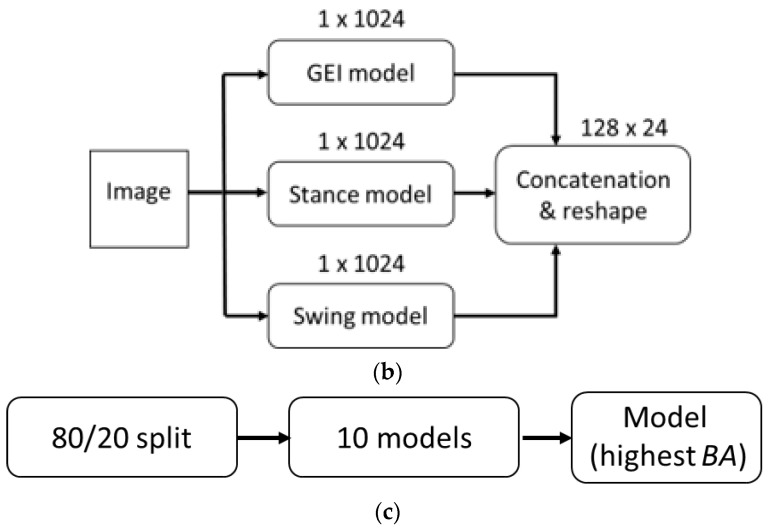
(**a**) Training of Stage 1 + Stage 2 CNNs using 80% training and 20% validation data; (**b**) Stage 1 CNN; and (**c**) Stage 2 CNN.

The three Stage 1 CNNs are fused and then joined with the Stage 2 CNN, as shown in Figure 2. As shown in Figure 11, the whole gait dataset is divided into an 80% training set and a 20% validation set. Training of the whole framework is repeated 10 times. Trained models with the highest balanced accuracy are further tested. With the same selection method as shown in Figure 10, the model with the highest balanced accuracy in the test set is finally used to perform gender classification on the whole gait dataset.

## 4. Experiments and Results

We train our proposed models on a computer with Intel i5 CPU, Nvidia GeForce RTX 3060 GPU, 32 GB RAM, and 1TB disk memory. To ensure compatibility with the model, each image is resized to the dimension of 105 × 226 pixels with bicubic interpolation over a 4 × 4 pixel neighborhood. We evaluate and compare the performance of our proposed framework with state-of-the-art gait-based gender classification methods on two benchmark datasets, CASIA B [39] and OU-ISIR MVLP [36]. Those recently proposed methods include a texture-based algorithm, posture-based algorithm, and deep learning-based model. El-Alfy et al. [13] proposed a texture-based gender recognition algorithm based on fuzzy local binary pattern (FLBP*) features extracted from GEIs. SVM is adopted for the prediction of the gender of the walking subject. Experimental results on the CASIA B dataset demonstrate that FLBP* outperforms four other LBP-based methods. Isaac et al. [41] proposed a posture-based gender classification method. Instead of demanding a complete gait cycle, they proposed a method that extracts features from each frame of the image sequence. Gender classification, called pose-based voting (PBV), is achieved based on the most probable predictions. Experimentations were performed on the CASIA B dataset with various feature extraction techniques, e.g., elliptic Fourier descriptors (PBV-EFD). Linear discriminant analysis (LDA) is adopted for gender classification. The two models achieve high gender classification accuracy, even surpassing the CNN+SVM method. Russel and Selvaraj [30] proposed a complex gender classification framework containing six parallel CNNs with the input of GEIs. The parallel networks contain varying numbers of convolution layers. Besides evaluating the performance of gender classification from multiple networks, they also investigate the performance of individual networks. For comparison, we select a single network model, CNN C_customized, which has similar complexity as our proposed models.

An investigation is performed on CASIA B [39] and OU-ISIR MVLP [36] datasets. CASIA [39] is a gait database with multi-view images. The CASIA B gait dataset consists of videos captured from 124 subjects. There are 110 video sequences captured at 11 view angles for each subject under 3 walking conditions (normal, with a bag, and in a coat). For the 10 video sequences per view angle, 6 are normal walking, 2 are carrying bags, and 2 are wearing coats. OU-ISIR MVLP contains videos captured by 14 cameras set in different view angles. There are two video sequences for each view angle. It is a large dataset involving 10,307 subjects. The dataset also provides a total of 267,386 pre-computed GEIs. Table 1 illustrates the details of these two datasets. Each image is resized to 105 × 226 pixels to be input into our proposed framework.

To evaluate the gender classification accuracy, we calculate the *Recall*, *Precision*, total accuracy (*Acc*), *F*1-*score*, and balanced accuracy (*BA*), where *TP* is true positive, *TN* is true negative, *FP* is false positive, *FN* is false negative, *TPR* is true positive rate, and *TNR* is true negative rate. *Recall* is the fraction of relevant classes predicted among all relevant classes in the dataset. *Precision* is the fraction of relevant classes predicted among all the predictions. *Recall* provides a measure of completeness, and *Precision* is a measure of exactness. *F*1-*score* provides a compact measure, which is a weighted harmonic mean of *Recall* and *Precision*. *Acc* is the fraction of correct predictions among all samples in the dataset. *BA* is the average of the true predictions. It provides an overall performance, whether or not the true labels are balanced in the dataset.

(4) $$Recall=\frac{TP}{TP+FN}$$

(5) $$Precision=\frac{TP}{TP+FP}$$

(6) $$Acc=\frac{TP+TN}{TP+FP+TN+FN}$$

(7) $$F1-score=\frac{2\times Precision\times Recall}{Precision+Recall}\;$$

(8) $$TPR=\frac{TP}{TP+FN}\;$$

(9) $$TNR=\frac{TN}{TN+FP}\;$$

(10) $$BA=\frac{TPR+TNR}{2}\;$$

Table 2 compares the performance of our proposed model (Stage 1 + Stage 2B) and three state-of-the-art methods on the CASIA B dataset in terms of *Recall*, *Precision*, *F*1-*score*, and *Acc*. The best result is highlighted in red. The second-best result is highlighted in blue. FLBP* extracts features from the GEI. PBV extracts features from the posture. Our proposed model outperforms these two methods with features from both GEIs and posture images. CNN C_customized also extracts features from the GEI. Although it achieves the highest *Precision*, the *F*1-*score* and *Acc* are relatively low due to the low value of *Recall*. Our proposed model achieves uniformly high values in *Recall*, *Precision*, *F*1-*score,* and *Acc*. As compared with the three recently proposed methods covering deterministic algorithms and the CNN model, our proposed method achieves the best score in *Recall*, *F*1-*score,* and *Acc* and the second-best score in *Precision*.

For more detailed gender classification results, we show the confusion matrix in Table 3. Most of the male and female samples are correctly predicted. Table 4 shows the gender classification result obtained by the GEI CNN with respect to the walking status. It illustrates the effectiveness of the feature extractor irrespective of other factors, such as walking status. Table 5 compares our proposed model with FLBP* with respect to the three walking statuses. Both methods achieve similar *Acc* for normal walking. For complicated statuses, our proposed model clearly outperforms FLBP*.

GEINet [22] is an eight-layered network that inputs GEIs into two triplets (convolution, pooling, and normalization) and two fully connected layers. GaitSet [26] is a flexible multi-stream CNN framework with the input of a gait silhouette sequence. Xu et al. [24] proposed a CNN framework. The model contains two GaitSet networks for two reconstructed gait silhouette sequences during training, while only one GaitSet network is used for testing. These deep learning-based methods exploit inputs of the GEI, a single original gait sequence, and multi-view synthesized gait sequences. Table 6 compares the performance of our proposed model and three methods as mentioned above on the OU-ISIR MVLP dataset in terms of *Acc*. Our proposed model outperforms all three reference methods. GaitSet, with the input of a large number of gait silhouettes in parallel, is a complex network. Our proposed model, which is a relatively simpler network with the inputs of the GEI and posture images, can perform better than Xu et al.’s [24] complex framework with two GaitSet networks.

For more detailed gender classification results, we show the confusion matrix in Table 7. Table 8 shows gender classification results obtained with respect to age group. It illustrates the capability of our proposed model in classifying gender in different age groups. The highest *Acc* is achieved in the largest age group of 16–60.

**Figure 12:**
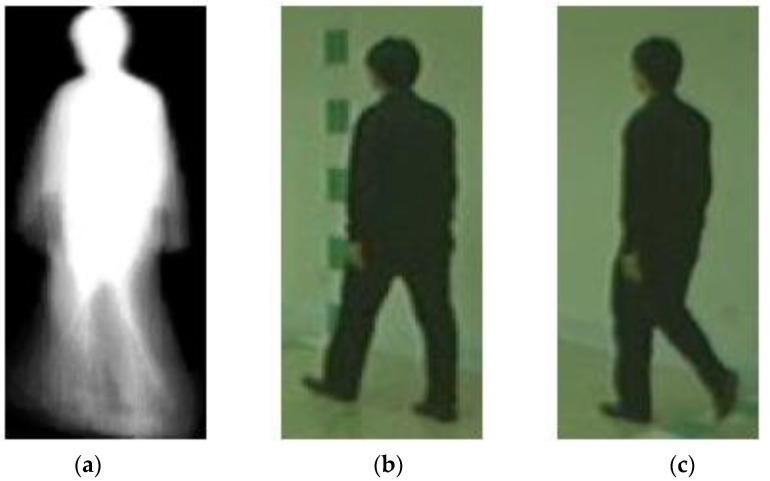
The set of input images that are correctly recognized. (**a**) GEI; (**b**) stance image; and (**c**) swing image.

**Figure 13:**
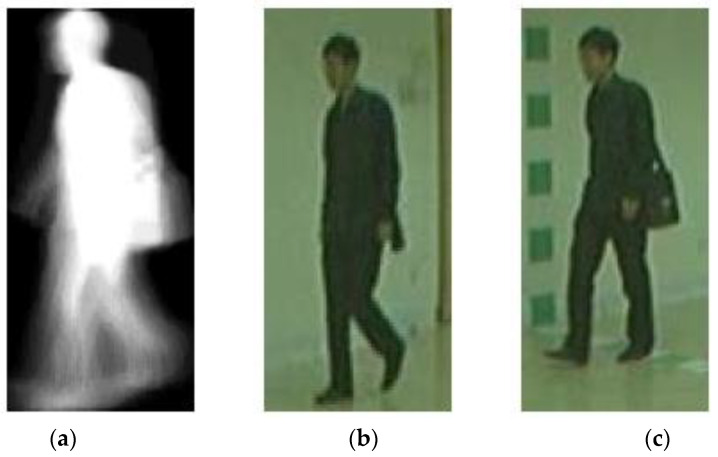
The set of input images that are wrongly recognized. (**a**) GEI; (**b**) stance image; and (**c**) swing image.

Figure 12 shows the set of input images which is correctly predicted by our proposed gender classification framework. The walking status is normal. Our proposed framework is capable of recognizing the gender of the subject correctly. However, a few viewing angles are more challenging and have more incorrect gender recognition results. For instance, the wrongly recognized data samples shown in Figure 13 are captured at a viewing angle of 54 degrees. Moreover, the human subject is carrying a bag. As presented in the numeric results, the accuracy of gender classification for this walking status is lower than that of normal walking.

## 5. Ablation Study

We evaluate and compare Stage 1 GEI CNN and our three proposed models based on *Recall*, *Precision*, *F*1-*score*, and *Acc*. Table 9 and Table 10 show the performance of all models on the CASIA B dataset and OU-ISIR-MVLP dataset, respectively.

The feature extractor network (three-stream Stage 1) has separate modules to process multi-modality inputs of GEIs and stance and swing images. The three modules are first trained independently. Features extracted by these three modules are fused and further analyzed in the classification network (Stage 2). The three proposed models achieve higher *Recall*, *Precision*, *F*1-*score*, and *Acc* than the single-stream Stage 1 GEI CNN on both datasets. It demonstrates that the proposed framework, which exploits features from both GEIs and postures, achieves higher gender classification accuracy than the model that only utilizes GEIs. On the CASIA B dataset, *Acc* is improved by 0.106 (10.6%). On the OU-ISIR MVLP dataset, *Acc* is improved by 0.133 (13.3%). The accuracies of the three proposed models are close. Based on our selection method, as mentioned previously, Stage 1 + Stage 2B is selected for CASIA B, while Stage 1 + Stage 2A is selected for OU-ISIR MVLP. Generally, results obtained from the CASIA B dataset are higher than those from the OU-ISIR MVLP dataset. The possible reason is that there are more varieties in gait due to the factor of age group in OU-ISIR MVLP, as compared with walking status in the CASIA B dataset.

We identify two hyperparameters—dropout rate and learning rate. Table 11 shows the performance of the Stage 1 + Stage 2C model with respect to the dropout rate based on area under the ROC curve (*AUC*). To optimize the performance of the Stage 1 + Stage 2 model, we measure *AUC* with a dropout rate varying from 0.1 to 0.7. As highlighted in Table 11, the optimal dropout rate is 0.5.

Table 12 shows the performance of the three proposed models with respect to the learning rate based on *AUC*. To optimize the performance of the three models, we measure *AUC* with a learning rate varying from 0.0008 to 0.0012. As highlighted in Table 12, the optimal learning rate is 0.0010 for all three proposed models.

Table 13 compares the inference time per one set of inputs (GEI, stance image, and swing image) and the number of parameters of our proposed models. Stage 1, a multi-stream CNN, contains most of the parameters of the framework. That guarantees the feature extractor has sufficient analytical power to extract useful features from the multiple modality inputs. The three proposed models demand a similar amount of time to produce classification results. In terms of model complexity, Stage 2A has more trainable parameters than Stage 2B and Stage 2C due to the larger number of nodes in the densely connected layer.

## 6. Conclusions

We propose a multi-stream cascade network for gender classification with heterogeneous inputs of GEIs and stance and swing postures. The framework contains the multi-stream feature extractor and gender classifier. The feature extractor is trained to gradually learn from the multiple input images. We adopt ensemble learning to train the cascade network. Based on the proposed framework, we design three gender classification models. We evaluate our proposed models and compare them with various gender classifier methods on two benchmark datasets. We demonstrate that the proposed framework with the additional features extracted from the stance/swing images achieves higher gender classification accuracy than a variety of recently proposed methods that only utilize a single type of input, such as the GEI or posture image.

In the future, we will continue our research on gait recognition. Besides gender classification, deep learning models can be trained for recognition of walking angle and walking status, as well as age estimation. As demonstrated in our numeric and visual results, the accuracy of gender classification for the walking status of “with a bag” or “in a coat” is lower than that of normal walking. The gender recognition model should be enhanced to address this limitation. As illustrated in Figure 13, some viewing angles are more challenging, and the gender of the subject may be wrongly recognized. Moreover, we will exploit other inputs. For instance, a deep learning model can be trained to generate the skeleton sequence from the video. Human gait can also be measured by micro-electro-mechanical system (MEMS) sensors [42]. Our multi-stream framework can be extended to accommodate additional input modalities. Other networks, e.g., 3D CNN, can be utilized to analyze the input of video sequences. A comprehensive gait recognition framework will be proposed for the provision of more predictions, e.g., age group, age, and ethnicity.

## Figures and Tables

**Table 1:** Details of CASIA B and OU-ISIR MVLP datasets.

Characteristics	CASIA B	OU-ISIR MVLP
Number of male subjects	93	5114
Number of female subjects	31	5193
Number of view angles	11	14
View range	0–180	0–90, 180–270
Age range	-	2–87
Image resolution	352 × 240	1280 × 980

**Table 2:** Comparison of our proposed gender classification model with other methods on CASIA B dataset.

Method	*Recall*	*Precision*	*F*1-*score*	*Acc*
FLBP* [13]	0.902	0.907	0.904	0.903
PBV-EFD [41]	-	-		0.953
CNN C_customized [30]	0.929	0.995	0.961	0.960
Our proposed model (Stage 1 + Stage 2B)	0.982	0.979	0.980	0.981

**Table 3:** Confusion matrix of our proposed gender classification model on CASIA B dataset.

Truth Predict	Female	Male	Total
Female	23,028	1047	24,075
Male	783	29,758	30,541
Total	23,811	30,805	54,616

**Table 4:** Gender classification result with respect to walking status on CASIA B dataset.

Walking Status	Female *Recall*	Female *Precision*	Female *F*1-*score*	Male *Recall*	Male *Precision*	Male *F*1-*score*	*BA*
Normal	0.972	0.971	0.972	0.980	0.981	0.981	0.976
With a bag	0.971	0.967	0.969	0.977	0.980	0.978	0.973
In a coat	0.950	0.953	0.952	0.968	0.966	0.967	0.960

**Table 5:** Comparison of our proposed gender classification model with other methods with respect to walking status on CASIA B dataset based on *Acc*.

Walking Status	FLBP* [13]	Our Proposed Model	Difference
Normal	0.964	0.963	−0.001
With a bag	0.880	0.961	+0.081
In a coat	0.865	0.941	+0.076

**Table 6:** Comparison of our proposed model with other methods on OU-ISIR MVLP dataset based on Acc.

Method	*Acc*
GEINet [22]	0.939
GaitSet [26]	0.927
Xu [24]	0.943
Our proposed model (Stage 1 + Stage 2A)	0.948

**Table 7:** Confusion matrix of our proposed model on OU-ISIR MVLP dataset.

Truth Predict	Female	Male	Total
Female	28,441	1730	30,171
Male	1336	27,923	29,259
Total	29,777	29,653	59,430

**Table 8:** Gender classification result with respect to age group on OU-ISIR MVLP dataset.

Age Group	Female *Recall*	Female *Precision*	Female *F*1-*Score*	Male *Recall*	Male *Precision*	Male *F*1-*Score*	*BA*
0–5	0.753	0.913	0.825	0.909	0.742	0.817	0.831
6–10	0.791	0.848	0.819	0.857	0.803	0.829	0.824
11–15	0.864	0.853	0.858	0.863	0.874	0.868	0.864
16–60	0.927	0.856	0.890	0.858	0.928	0.892	0.893
Over 60	0.893	0.640	0.746	0.681	0.909	0.779	0.787

**Table 9:** Performance evaluation of Stage 1 CNN and Stage 1 + Stage 2 CNN on CASIA B dataset.

Model	Female *Recall*	Female *Precision*	Female *F*1-*Score*	Male *Recall*	Male *Precision*	Male *F*1-*Score*	*Acc*
Single-stream Stage 1 (only GEI CNN)	0.870	0.881	0.875	0.879	0.869	0.874	0.875
Three-stream Stage 1 + Stage 2A	0.970	0.980	0.975	0.984	0.976	0.980	0.978
Three-stream Stage 1 + Stage 2B	0.992	0.964	0.978	0.973	0.994	0.983	0.981
Three-stream Stage 1 + Stage 2C	0.977	0.964	0.970	0.972	0.982	0.977	0.974

**Table 10:** Performance evaluation of Stage 1 CNN and Stage 1 + Stage 2 CNN on OU-ISIR MVLP dataset.

Model	Female *Recall*	Female *Precision*	Female *F*1-*Score*	Male *Recall*	Male *Precision*	Male *F*1-*Score*	*Acc*
Single-stream Stage 1 (only GEI CNN)	0.844	0.778	0.810	0.789	0.852	0.819	0.815
Three-stream Stage 1 + Stage 2A	0.955	0.943	0.949	0.942	0.954	0.948	0.948
Three-stream Stage 1 + Stage 2B	0.942	0.947	0.944	0.945	0.940	0.942	0.943
Three-stream Stage 1 + Stage 2C	0.944	0.946	0.945	0.944	0.942	0.943	0.944

**Table 11:** Performance evaluation of Stage 1 + Stage 2C model with varying dropout rate on CASIA B dataset.

Dropout Rate	*AUC*
0.1	0.961
0.2	0.961
0.3	0.961
0.4	0.965
0.5	0.967
0.6	0.953
0.7	0.966

**Table 12:** *AUC* of the three proposed models with varying learning rates on CASIA B dataset.

Learning Rate	Stage 1 + Stage 2A	Stage 1 + Stage 2B	Stage 1 + Stage 2C
0.0008	0.976	0.965	0.961
0.0009	0.970	0.970	0.955
0.0010	0.982	0.972	0.974
0.0011	0.978	0.957	0.971
0.0012	0.977	0.968	0.969

**Table 13:** Inference time per single set of GEI/stance/swing images and number of parameters of our proposed models.

Model	Inference Time (s)	Number of Parameters
Stage 1	0.029	52 M
Stage 1 + Stage 2A	0.305	62 M
Stage 1 + Stage 2B	0.327	53 M
Stage 1 + Stage 2C	0.308	53 M

## Data Availability

Data sharing is not applicable.

## Appendix A

The following list illustrates the 55 training sets, each of which is formed by combining data samples from two viewing angles. The first column shows the two viewing angles to be joined as one training set. For instance, 0° + 18° is the set containing inputs from viewing angles of 0 and 18 degrees. The second column shows the size of the training set.

**Table A1:** Training sets generated from combination of two viewing angles.

Training Set	Number of Samples
0° + 18°	3716
0° + 36°	3691
0° + 54°	3704
0° + 72°	3695
0° + 90°	3684
0° + 108°	3727
0° + 126°	3678
0° + 144°	3679
0° + 162°	3700
0° + 180°	3705
18° + 36°	3717
18° + 54°	3730
18° + 72°	3721
18° + 90°	3710
18° + 108°	3753
18° + 126°	3704
18° + 144°	3705
18° + 162°	3726
18° + 180°	3731
36° + 54°	3705
36° + 72°	3696
36° + 90°	3685
36° + 108°	3728
36° + 126°	3679
36° + 144°	3680
36° + 162°	3701
36° + 180°	3706
54° + 72°	3709
54° + 90°	3698
54° + 108°	3741
54° + 126°	3692
54° + 144°	3693
54° + 162°	3714
54° + 180°	3719
72° + 90°	3689
72° + 108°	3732
72° + 126°	3683
72° + 144°	3684
72° + 162°	3705
72° + 180°	3710
90° + 108°	3721
90° + 126°	3672
90° + 144°	3673
90° + 162°	3694
90° + 180°	3699
108° + 126°	3715
108° + 144°	3716
108° + 162°	3737
108° + 180°	3742
126° + 144°	3667
126° + 162°	3688
126° + 180°	3693
144° + 162°	3689
144° + 180°	3694
162° + 180°	3715
